# Personality dysfunction in opiate addicts on opioid substitution treatment and the risk of HCV infection

**DOI:** 10.3389/fpubh.2022.1009413

**Published:** 2022-09-09

**Authors:** Siniša Skočibušić, Nera Zivlak-Radulović, Mevludin Hasanović, Hassan Awad, Dragana Karan-Križanac, Nermana Mehić-Basara, Tomislav Rukavina

**Affiliations:** ^1^Clinic for Infectious Diseases, University Clinical Hospital Mostar and Center for Prevention and Outpatient Treatment of Addiction, Mostar, Bosnia and Herzegovina; ^2^Clinic for Psychiatry, University Clinical Center of Republic of Srpska, Banja Luka, Bosnia and Herzegovina; ^3^Clinic for Psychiatry, University Clinical Center Tuzla, Tuzla, Bosnia and Herzegovina; ^4^Public Institute for Addiction Disorders of Canton Zenica-Doboj, Zenica, Bosnia and Herzegovina; ^5^Department of Pathology, Cytology and Forensic Medicine, University Clinical Hospital Mostar, Mostar, Bosnia and Herzegovina; ^6^Public Institute for Addiction Disorders of Canton Sarajevo, Sarajevo, Bosnia and Herzegovina; ^7^Faculty of Medicine, University of Rijeka and Teaching Institute of Public Health, Rijeka, Croatia

**Keywords:** opiate substitution treatment, hepatitis C, chronic, risk factors, personality disorders, opiate addicts

## Abstract

**Background:**

Impulsivity, affective instability, and neglect of oneself and other people's safety as symptoms of personality dysfunction are associated with risky behaviors regarding the transmission of infectious diseases either sexually or by intravenous drug abuse.

**Objective:**

The aim of this study was to analyze the association between hepatitis C virus (HCV) infection and personality dysfunction in opiate addicts on opioid substitution treatment.

**Methods:**

This was a cross-sectional, observational investigation of patients over 18 years of age who were actively participating in opioid substitution treatment at five centers in Bosnia and Herzegovina. The occurrence of HCV infection was the primary study outcome, and personality functioning, the main independent variable, was assessed using the Severity Indices of Personality Problems (SIPP−118) questionnaire. The association between scores of personality functioning domains items and HCV infection status was determined by binary logistic regression analysis.

**Results:**

Patients on opioid substitution therapy with HCV infection more frequently had personality disorders (OR 2.168, 95% CI 1.161–4.05) and were treated longer than patients without HCV infection (OR 1.076, 95% CI 1.015–1.14). HCV infection was associated with lower self-respect (OR 0.946, 95% CI 0.906–0.988), decreased capacity to have enduring relationships with other people (OR 0.878, 95% CI 0.797–0.966), and lower capability to cooperate with others (OR 0.933, 95%CI 0.888–0.98). On the other hand, except for self-respect, other elements of the Identity Integration domain (enjoyment, purposefulness, stable self-image, and self-reflexive functioning), when more functional, increased the risk of HCV infection.

**Conclusions:**

Our study demonstrates that opiate addicts on opioid substitution treatment have a higher risk of HCV infection if their personality is dysfunctional, especially in the aspects of self-respect, enduring relationships, and cooperativity. The risk is even higher in addicts who have an established diagnosis of any kind of personality disorder.

## Introduction

The connection between personality and somatic diseases has long been present in the medical literature; however, until recently, there was not much evidence to confirm the existence of concrete examples. In the last decade, it was recognized that severe psychological traumas, which cause personality disorders, cause both somatoform and some somatic diseases (1). The extent to which personality dysfunction is a necessary link between psychological trauma and somatic illness is still unclear; however, there is much current research addressing this issue in various fields of medicine. It has recently been shown that personality disorders related to obesity, accompanied by a large number of comorbidities, are also associated with personality functioning (2). The connection between personality disorders and infections is particularly interesting. In a study by Scheidell et al., it was shown that personality disorders increase the risk of sexually transmitted infections, especially the human immunodeficiency virus (HIV) infection (3). It has been observed that impulsivity, affective instability, and neglect of oneself and other people's safety as symptoms of personality dysfunction have been associated with risky behaviors regarding sexually transmitted diseases, such as condom-free sex in women and condom-free oral sex in women and men (4). A connection between risky behavior and prevalence of hepatitis C virus (HCV) infection was reported by Handanagić et al. in Croatia (5).

In the psychiatric literature, a distinction is made between personality disorders (of which there are ten main types with their own special characteristics) and the degree to which a person can adapt to the environment (6). A person may have a certain personality disorder (e.g., obsessive-compulsive-type disorder) and be very adaptable, especially in certain situations (e.g., in situations that require exceptional achievement at work). Thus, there is no ideal inverse correlation between the severity of personality disorders (the degree of expression of characteristic traits) and the degree of adjustment (adaptation) of personality functioning (7). Therefore, the degree of adjustment of personality functioning is a special general entity that exists in groups of individuals with personality disorders and normal people; as such, it can be measured separately.

In subjects on opioid substitution treatment, very often (60.1%), behavioral disorders in childhood (conduct disorders) could be found, which are considered prodromes of personality disorders and problems with personality functioning (8). One type of personality disorder (the antisocial type) was found in as many as 14.7% of patients on opioid substitution treatment at a Malaysian university hospital (9). On the other hand, the prevalence of HCV infection among patients on opioid substitution treatment is high, varying from 70 to 72.51% (10, 11). Although it has been previously shown that personality disorders are risk factors for HCV infection, e.g., in prisons (11, 12), it remains uncertain whether personality malfunction is a risk factor for HCV infection and, if such a link exists, whether it is working in a subpopulation of opioid substitution treatment subjects regardless of whether or not the patients were diagnosed with personality disorders. Also, genotype 1 HCV replicates more efficiently, damages the liver more severely, and infection with it has a worse prognosis than infection with other genotypes (13). The severity of the disease certainly affects the quality of life and further complicates the adjustment of the patient's personality to external requirements; so, it is interesting to examine whether people on opioid substitution treatment having personality malfunction are at higher risk of infection with this genotype than others.

The aim of this study was to analyze the association between HCV infection and personality dysfunction in opiate addicts on opioid substitution treatment.

## Methods

The study was designed as a cross-sectional, observational investigation. The inclusion criteria were as follows: age over 18 years, participation in opioid substitution treatment for at least 3 months, and provision of written informed consent to participate in the research. The exclusion criteria were as follows: presence of malignant tumors, presence of major mental or neurological disorders (schizophrenia, cognitive disorders, mental retardation, etc.), and pregnancy. The study sample was of the convenient type, capturing all available subjects treated in five centers for opioid substitution in Bosnia and Herzegovina in 2019–2021 at the following healthcare facilities: Clinic for Psychiatry, University Clinical Center of Republic of Srpska, Banja Luka; Center for Prevention and Outpatient Treatment of Addiction Mostar, Mostar; Institute for Addiction Diseases of Sarajevo Canton, Sarajevo; Institute for Addiction Diseases of Zenica-Doboj Canton, Zenica; Clinic for Psychiatry; University Clinical Center Tuzla, Tuzla. The study was approved by the ethics committees of the involved healthcare facilities.

The primary study outcome was HCV infection; it was tested in laboratories at the study sites first *via* serological methods (detection of anti-HCV antibodies) and confirmed *via* molecular methods based on a combination of amplification methods (polymerase chain reaction, PCR, which allows for detection of HCV in the blood) and detection methods (hybridizations with HCV-specific assays labeled with chromogenic or fluorescent dyes, which enable the quantification of viremia) (14, 15). The genotypes of the virus (1–6) in each subject were determined *via* the same methods.

Potential independent variables and confounders (general, demographic, social, and health variables) in opiate addicts relevant to this study were examined using the standardized questionnaire, “Treatment Requirement Indicator 3.0” (TDI 3.0) (16). The existence of a diagnosis of a personality disorder (of any type) was determined by inspecting the medical records of the study subjects. Among the potential predictors of HCV infection were scores of the Severity Indices of Personality Problems (SIPP−118) questionnaire (6). SIPP 118 is the full version of the questionnaire, while SIPP SF is an abbreviated version intended primarily to monitor the effects of treatment. Both versions of the questionnaire are used to measure the degree of adjustment of personality functioning not only in people diagnosed with personality disorders but also in healthy ones. Based on the questionnaire, a T-score ranging from 0 to more than 70 is determined for each respondent. The higher the score, the more adjusted the functioning of the personality. The limit below which the subject is considered to be in the category of personality dysfunction is lower for the healthy population (T-score 30) than for the population of respondents with personality disorders (T-score 35). The questionnaire has five domains: self-control, identity integration, accountability, ability to establish relationships with others, and ability to cooperate with others. In the Dutch population, it has been validated for both adults and adolescents (17), while in the Croatian population it has been validated for adults (18). Consent to use the Croatian-translated version in this study was obtained from both the author of the Croatian validation study and the author of the original questionnaire in English (the consent form was signed by Mrs. Laura Weekers on 20 February 2017).

Data processing was carried out first *via* descriptive statistical methods. The normality of the sample distribution of continuous variables was analyzed by Kolmogorov-Smirnov test. Data are presented as frequencies and percentages for categorical variables, as means ± standard deviations for normally distributed continuous variables, and as medians (with interquartile ranges) for continuous variables with skewed data distributions. Chi-square test, Student's *t*-test for independent samples, one-way analysis of variance for normal data distributions, and Mann-Whitney U test or Kruskal-Wallis nonparametric analysis of variance for skewed data distributions were conducted to test the difference between groups. Binary logistic regression analyses were performed to determine predictive values of risk factors for HCV infection. A stepwise regression elimination procedure was used to select variables to be included in the regression model. A *p*-value of <0.05 was considered statistically significant. SPSS for Windows (version 20.0, SPSS Inc., Chicago, IL, United States) was used for statistical analyses.

## Results

A total of 206 opioid-dependent patients on the opioid-substitution program participated in the study, of whom 19 were women and 187 were men. The median of the study participants was 29 years, and the interquartile range was 10 years. The subjects were classified into two groups according to the presence (*n* = 120) or absence (*n* = 86) of HCV infection. The characteristics of patients in the two groups are shown in Table 1.

**Table 1:** Characteristics of patients according to HCV status.

**Characteristics**	**HCV-positive patients (mean ±SD, median, IQR)**	**HCV-negative patients (mean ±SD, median, IQR)**	**Null hypothesis probability (*p*)**
Age (years)	28.9 ± 6.8, 29.0 [10.0], *n =* 120	30.1 ± 8.7, 29.0 [12.3], *n =* 86	0.484
Sex (m/f)	107/13 (89.2/10.8%)	80/6 (93.0/7.0%)	0.345
Duration of substance dependency therapy (years)	9.3 ± 5.6, 10.0 [7.8], *n =* 120	7.3 ± 5.1, 7.0 [8.3], *n =* 86	0.021
Age of the first substance abuse (years)	20.0 ± 5.4, 19.0 [7.0], *n =* 120	21.5 ± 6.1, 20.0 [8.0], *n =* 86	0.088
Age of opioid substitution onset (years)	28.8 ± 6.4, 28.5 [9.8], *n =* 120	30.4 ± 8.5, 29.0 [13.0], *n =* 86	0.295
Age of the first intravenous substance abuse (years)	22.3 ± 5.9, 20.0 [8.0], *n =* 98	23.7 ± 5.6, 23.0 [7.8], *n =* 48	0.077
Viral load (copies/ml)	2,974,130 ± 6,281,029, 837,531 [3,108,176], *n =* 84	NA	NA
Type of treatment facility (outpatient/hospital/prison)	64/55/1 (53.3/45.8/0.8%)	58/28/0 (67.4/32.6/0%)	0.137
Living: alone/with primary family/with partner/else	22/74/21/3 (18.3/61.7/17.5/2.5%)	14/51/21/0 (16.3/59.3/24.4/0%)	0.587
Children: none/living with children/not living with children/not known	69/28/19/4 (57.5/23.3/15.8/3.4%)	51/19/13/3 (59.3/22.1/15.1/3.5%)	0.996
Lodging: stable/unstable or homeless	111/9 (92.5/7.5%)	82/4 (95.3/4.7%)	0.863
Employment: occasional/regular/student/unemployed/other	30/22/0/55/13 (25.0/18.3/0.0/45.8/10.8%)	22/20/1/41/2 (25.6/23.3/1.2/47.7/2.3%)	0.164
Education: university/high school/elementary school or less	6/86/28 (5.0/71.7/23.3%)	4/62/20 (4.7/72.1/23.2%)	0.838
Primary substance of abuse: heroin/other opioids/cocaine/amphetamines/unknown	97/0/0/0/23 (80.8/0/0/0/19.2%)	69/4/1/3/9 (80.2/4.7/1.2/3.5/10.5%)	0.004
Route of administration: injection/smoking/snorting/oral/other	97/10/12/0/1 (80.8/8.3/10.0/0/0.8%)	37/16/22/10/1 (43.0/18.6/25.6/11.6/1.2%)	0.000
Rate of abuse: daily/4–6 days weekly/2–3 days weekly/once weekly or less/not used in the last 30 days/not known	41/1/6/7/63/2 (34.2/0.8/5/5.8/52.5/1.7%)	32/2/6/8/37/1 (37.2/2.3/7/9.3/43/1.2%)	0.669
Abuse of more than one substance: yes/no	74/46 (61.7/38.3%)	53/33 (61.6/38.4%)	0.995
Previous opioid substitution: yes/no/not known	75/44/1 (62.5/36.7/0.8%)	55/31/0 (64/36/0%)	1.000
Diagnosed with personality disorder: yes/no	69/51 (57.5/42.5%)	32/54 (37.2/62.8%)	0.004

In terms of the value of individual domains of the SIPP 118 questionnaire (which measures the severity of personality dysfunction), the HCV-positive patients differ from the HCV-negative patients only in terms of self-esteem, which was significantly lower (*p* =0.021). All other aspects of personality functioning were similarly assessed in the HCV-positive and HCV-negative patients (Table 2).

**Table 2:** Values of SIPP 118 items and domains according to HCV infection status.

**SIPP 118 items and domains**	**HCV-positive patients (mean ±SD, median, IQR)**	**HCV-negative patients (mean ±SD, median, IQR)**	**Null hypothesis probability (*p*)**
Self-control domain	55.9 ± 10.9, 55.6 [15.4]	57.6 ± 12.0, 57.8 [20.9]	0.310
Identity integration domain	63.6 ± 12.3, 64.0 [17.7]	66.4 ± 11.1, 66.9 [18.0]	0.152
Responsibility domain	47.1 ± 9.7, 46.8 [13.8]	49.3 ± 10.9, 49.6 [18.2]	0.130
Relational capacity domain	59.1 ± 9.1, 59.7 [13.0]	60.1 ± 8.7, 60.2 [15.4]	0.562
Social concordance domain	54.3 ± 10.6, 55.9 [16.4]	55.5 ± 10.8, 57.7 [18.9]	0.403
Self-control domain—emotional regulation item	56.8 ± 10.6, 57.0 [14.9]	58.1 ± 11.5, 59.1 [21.3]	0.484
Self-control domain—effortful control item	50.5 ± 9.2, 49.3 [13.9]	52.2 ± 9.9, 52.3 [13.9]	0.159
Identity integration domain—self-respect	62.2 ± 10.2, 63.2 [15.6]	65.4 ± 9.5, 67.1 [15.7]	0.021
Identity integration domain—stable self-image	59.4 ± 11.1, 58.8 [15.3]	62.3 ± 11.0, 63.3 [18.7]	0.077
Identity integration domain—self-reflexive functioning	57.1 ± 10.0, 57.1 [15.6]	58.1 ± 10.9, 58.2 [17.9]	0.598
Identity integration domain—enjoyment	59.1 ± 10.8, 59.3 [19.5]	61.3 ± 9.1, 61.6 [13.4]	0.253
Identity integration domain—purposefulness	59.7 ± 10.9, 60.0 [15.2]	61.3 ± 9.7, 63.3 [15.8]	0.351
Responsibility domain—responsible industry	47.1 ± 9.7, 45.0 [13.0]	49.1 ± 11.0, 49.3 [17.4]	0.116
Responsibility domain—trustworthiness	47.0 ± 9.8, 45.9 [12.3]	49.2 ± 10.7, 50.0 [14.9]	0.106
Relational capacity domain—intimacy	54.7 ± 7.7, 52.8 [11.9]	53.6 ± 8.2, 53.8 [13.9]	0.347
Relational capacity domain—enduring relationship	61.3 ± 8.8, 62.5 [13.2]	63.1 ± 8.7, 63.6 [15.4]	0.190
Relational capacity domain—feeling recognized	53.0 ± 10.5, 52.7 [15.1]	52.8 ± 11.1, 53.8 [17.3]	0.851
Social concordance domain—aggression regulation	48.3 ± 9.8, 50.1 [18.9]	49.6 ± 11.3, 54.6 [18.5]	0.337
Social concordance domain—frustration tolerance	62.4 ± 11.7, 63.1 [16.5]	62.8 ± 12.8, 63.1 [18.8]	0.677
Social concordance domain—cooperation	54.2 ± 10.6, 53.9 [19.1]	56.5 ± 10.9, 56.0 [19.1]	0.108
Social concordance domain—respect	54.4 ± 9.8, 55.2 [13.3]	55.2 ± 9.6, 55.2 [15.9]	0.598

Comparisons of individual domains and elements of the SIPP 118 questionnaire in a subgroup of patients diagnosed with personality disorders (*n* = 101) according to the presence (*n* = 69) or absence (*n* = 32) of HCV infection found no significant differences. On the other hand, in the subgroup of patients who were not diagnosed with personality disorders (*n* = 105), the difference between those who were HCV-positive (*n* = 51) and HCV-negative (*n* = 54) was found in the self-esteem aspect. The mean HCV score of the HCV-positive patients was significantly lower (62.6 ± 10.1) than that of the HCV-negative patients (66.7 ± 9) according to the Mann-Whitney U test (U = 1,729.5, *p* = 0.024).

There was no significant difference in SIPP 118 questionnaire scores between the HCV-positive patients infected with genotype 1 and patients infected with other HCV genotypes.

To examine the potential predictors of HCV infection after adjusting for other relevant factors (gender, education, employment status, family status, diagnosis of personality disorder, length of treatment, age of the patient when starting addiction treatment, start of opioid supplementation, duration of treatment, age when opioid abuse was first started, and scores in SIPP 118 domains and items), a binary logistic regression model was built using a stepwise regression elimination procedure. The final model included nine variables (chi-square = 33.971, df = 8, *p* = 0). The Hosmer-Lemeshow goodness-of-fit test confirmed that the model described the data satisfactorily (*p* = 0.183), and the Cox and Snell R-square and Nagelkerke R-square values were 0.152 and 0.205, respectively. The potential predictors of HCV infection are shown in Table 3.

**Table 3:** Potential predictors of HCV infection.

**Predictor**	**Adjusted odds ratio**	**95% Confidence Interval**	***p*** **-value**
Relational Capacity SSIP domain	1.203	1.067–1.356	0.003
Identity Integration domain—Self-respect	0.946	0.906–0.988	0.012
Relational capacity domain—Enduring relationships	0.878	0.797–0.966	0.008
Social concordance domain—Cooperation	0.933	0.888–0.980	0.005
Duration of treatment	1.076	1.015–1.140	0.013
Any type of personality disorder	2.168	1.161–4.050	0.015

## Discussion

Our study showed that the patients with HCV infection on opioid substitution therapy more frequently had personality disorders, and that they were treated longer than the patients without HCV infection. HCV infection was associated with lower self-respect, decreased capacity to have enduring relationships with other people, and lower capability to cooperate with others. On the other hand, except for self-respect, the other elements of the Identity Integration domain (enjoyment, purposefulness, stable self-image, and self-reflexive functioning), when more functional, increased the risk of HCV infection.

Impulsivity is a characteristic of several types of personality disorder; however, it is especially prominent in the borderline type (19). The link between impulsivity and increased risk of HCV infection was already demonstrated when a sample of HCV-positive adult patients was compared with a sample of patients with human T-cell lymphotropic virus type 1 (HTLV-1) infection: The HCV-positive patients showed higher degrees of impulsivity and risky behavior (20). Borderline personality disorder among young adults was significantly associated with greater probability of syringe sharing, a well-known risk factor for HCV infection (21). Obviously, many traits of personality disorders make the subjects less cautious, less analytical, and more prone to disregard mechanisms of HCV transmission, which requires close contact and exchange of blood or bodily fluids. Besides, psychiatric patients with a significant physical co-morbidity, such as HCV infection, are more frequently re-admitted to hospitals and treated much longer than other patients (22), which can explain the association between the duration of treatment and the presence of HCV infection that was observed in our study.

It is not surprising that low self-respect was associated with higher risk of HCV infection. In a study on 418 HCV-positive injection drug users, it was demonstrated that low self-respect contributed to injection-related risk behaviors such as needle- or syringe-lending or sharing drug preparation equipment (23). The association between low self-esteem and risky sexual behavior was found in Nigerian adolescents: subjects with low self-esteem were 1.7 times more likely to engage in sexual activities; therefore, they were more likely to contract HCV infection than those with higher self-esteem (24). A survey conducted among college students revealed that those with higher self-esteem, who consumed less alcohol or did not drink it at all, and more often used condoms when engaging in sexual activities with partners they did not know well had a lower risk of contracting HCV infection (25).

Enduring relationships between younger adults (especially when partners are confident in the relationship) protect against types of sexual behaviors that come with a high risk of contracting HCV or other sexually transmitted infections (26). Within enduring relationships, there is more chance for psychological bonding and sustenance to be developed between the partners involved, which helps drug-dependent people to decrease their sense of social isolation and see meaning in life, Having such feelings, a drug-dependent person is more prone to quit the dependence or at least to avoid intravenous injection practices that increase the risk of contracting HCV infection (27). Our study confirmed these findings as the ability to achieve more enduring interpersonal relationships was associated with the HCV-negative status of the study participants. Social relations by themselves, if devoid of emotional and rational depth, may rather promote risky behavior and help establish alliances in substance abuse practices, consequently increasing the risk of HCV infection. This is a plausible explanation for our findings that higher values in the Relational Capacity domain of the SIPP 118 questionnaire were associated with HCV infection.

Social exclusion and uncooperative personality traits are associated with higher risk of HCV infection, as was observed in the Roma community in Brno, Czech Republic, whose members engaged in risky behaviors (28). The uncooperative behavior among drug addicts is one of the main reasons for the missed diagnosis of HCV infection, even when they are targeted *via* special programs, as experienced by physicians who run an outpatient addiction treatment clinic (29). Uncooperativeness is also a reason why drug addicts diagnosed with HCV infection rarely proceed with subsequent investigations or treatment (30). Patients in the opioid-substitution program in our study who were more cooperative were less frequently HCV-positive, and this finding identified cooperativeness as a personality functioning aspect that may protect against HCV infection.

There are several limitations to our study. First, the study sample was relatively small, precluding the identification of more subtle differences in personality functioning between the HCV-positive and HCV-negative study participants. Second, the participants were not randomly selected from the target population, which could have introduced a selection bias that is difficult to control with available study settings. Finally, there are other available instruments or methods for measuring personality functioning that, if used, could have given a more complete insight into the link between personality dysfunction types and risk of HCV infection.

Our study has shown that opiate addicts on opioid substitution treatment have a higher risk of HCV infection if their personality is dysfunctional, especially in the aspects of self-respect, enduring relationships, and cooperation. The risk is even higher in addicts who have an established diagnosis of any kind of personality disorder.

## Data availability statement

The raw data supporting the conclusions of this article will be made available by the authors, without undue reservation.

## Ethics statement

The studies involving human participants were reviewed and approved by Center for Prevention and Outpatient Treatment of Addiction, Mostar, Bosnia and Herzegovina: No 01-54/17; Public Institute for Addiction Disorders of Canton Sarajevo, Sarajevo, Bosnia and Herzegovina: No 0202-489/17; University Clinical Center of Republic of Srpska, Banja Luka, Bosnia and Herzegovina: No 01-9-339-2/17; University Clinical Center Tuzla, Tuzla, Bosnia and Herzegovina: No 02-09/2-55/17; and Public Institute for Addiction Disorders of Canton Zenica-Doboj, Zenica, Bosnia and Herzegovina: No 05-34-304-2/17. The patients/participants provided their written informed consent to participate in this study.

## Author contributions

SS designed the study. SS, NZ-R, MH, HA, and NM-B contributed to the implementation of the study. SS and DK-K wrote the draft of the manuscript. NM-B and TR helped with data analyses and supervised the study. All authors discussed the results and commented on the manuscript.

## Conflict of interest

The authors declare that the research was conducted in the absence of any commercial or financial relationships that could be construed as a potential conflict of interest. The reviewer NS declared a shared affiliation with one of the author MH to the handling editor at time of review.

## Publisher's note

All claims expressed in this article are solely those of the authors and do not necessarily represent those of their affiliated organizations, or those of the publisher, the editors and the reviewers. Any product that may be evaluated in this article, or claim that may be made by its manufacturer, is not guaranteed or endorsed by the publisher.
